# Diversity, structure and demography of coral assemblages on underwater lava flows of different ages at Reunion Island and implications for ecological succession hypotheses

**DOI:** 10.1038/s41598-020-77665-z

**Published:** 2020-11-30

**Authors:** Florian Jouval, Lionel Bigot, Sophie Bureau, Jean-Pascal Quod, Lucie Penin, Mehdi Adjeroud

**Affiliations:** 1 Université de La Réunion, UMR 9220 ENTROPIE, Faculté des Sciences et Technologies, 97744 Saint Denis Cedex 9, La Réunion France; 2 Laboratoire d’Excellence “CORAIL”, Paris, France; 3 Agence pour la Recherche et la Valorisation Marines (ARVAM-Pareto), Technopole de la Réunion, BP 80041, 97491 Sainte Clotilde, La Réunion France; 4 Institut de Recherche pour le Développement, UMR 9220 ENTROPIE, CRIOBE/UPVD, Avenue Paul Alduy, 66860 Perpignan, France

**Keywords:** Community ecology, Population dynamics

## Abstract

Understanding colonization of new habitats and ecological successions is key to ecosystem conservation. However, studies on primary successions are scarce for reef-building corals, due to the rarity of newly formed substratum and the long-term monitoring efforts required for their long life cycle and slow growth rate. We analysed data describing the diversity, structure and demography of coral assemblages on lava flows of different ages and coral reefs at Reunion Island, to evaluate the strength and mechanisms of succession, and its agreement to the theoretical models. No significant differences were observed between the two habitats for most structure and demographic descriptors. In contrast, species richness and composition differentiated coral reefs from lava flows, but were not related to the age of the lava flow. We observed a strong dominance of *Pocillopora* colonies, which underline the opportunistic nature of this taxa, with life-history traits advantageous to dominance on primary and secondary successional stages. Although some results argue in favor of the tolerance model of succession, the sequences of primary successions as theorized in other ecosystems were difficult to observe, which is likely due to the high frequency and intensity of disturbances at Reunion, that likely distort or set back the expected successional sequences.

## Introduction

Ecological succession is a fundamental process in the structure and dynamics of ecosystems, notably for recovery trajectories following disturbances^1–3^. Ecological succession was defined by Odum^4^ as the reasonably predictable process of community development to a stabilized climax stage. When environmental conditions are not undergoing major changes, this process is expected to increase the biological control of the environment and lead to a stabilized ecosystem^5^. However, ecological succession may be interrupted in ecosystems frequently impacted by large-scale disturbances or local stressors, thus preventing the attainment of such stable climax communities^1,2,6^. In fact, in highly diverse ecosystems such as coral reefs and tropical rain forests, community structure, diversity and successional trajectories are largely controlled by disturbances^7–9^ that may shift communities to alternative, multiple “stable” states^10–12^. Two major types of ecological succession have been distinguished. Primary succession characterizes the colonization of a newly formed environment (i.e., sterile in its origin), such as lava flows, by pioneer species characterized by small body sizes, high dietary flexibility and high reproductive rates^13^. Secondary succession refers to the sequential replacement of biota following a disturbance, such as forest fires or cyclones^14,15^. Connell and Slatyer^1^ have proposed three major models of ecological succession. The "facilitation" model implies that the colonization and growth of the later species is dependent upon the pioneer species "preparing the ground", and being progressively replaced by mid- and late successional species; only after this can later species colonize. The "tolerance" model suggests that a predictable sequence is produced by the existence of species that have evolved different strategies for exploiting resources. Later species will be those able to tolerate lower levels of resources than earlier ones. Thus, they can invade and grow to maturity in the presence of those that preceded them. The "inhibition" model suggests that all species resist invasions of competitors. The first occupants preempt the space and will continue to exclude or inhibit later colonists until the former die or are damaged, thus releasing resources and allowing later colonists to reach maturity. Although successional theory has been mainly developed for terrestrial ecosystems, where long-term chronological sequences are available and field experiments easier to implement^3,16^, it has been shown that some theoretical approaches developed in terrestrial ecology can be applied to marine ecosystems such are coral reefs (e.g., the “animal forest” concept^17^). In fact, the increasing threats to marine ecosystems call for further investigations on the extent of predictable and orderly changes of marine communities^2,9^, which can be addressed by examining the theoretical assumptions of Connell and Slatyer^1^.

Coral reefs are characterized by high biodiversity and complex biotic interactions, and provide critical ecosystem services for hundreds of millions of people^18–20^. Like many other ecosystems, coral reefs are facing increasing frequency and intensity of natural and anthropogenic perturbations of various origin^21–23^. These perturbations have caused widespread mortality of Scleractinian corals, the primary framework builders and key components of reef health and biodiversity, and have transformed the structure and dynamics of coral assemblages worldwide^9,24,25^, with potential phase-shifts from coral to macroalgal dominance^11,12,26^. Secondary succession processes have been widely studied in coral reef ecosystems through changes in community organization following large-scale disturbances, such as thermally induced coral bleaching events, cyclones and the sudden changes in abundance of keystone species such as herbivores and corallivores^25,27–29^. Most notably, successional dynamics have been well documented for algal communities, which are characterized by their ability for rapid colonization of vacant substrate, their short life cycle and fast growth rates^30–32^. In contrast, primary succession processes remain poorly investigated in coral reef ecosystems, particularly for Scleractinian corals. This lack of information is mainly related to the rarity of newly formed substrate that corals may colonize, and to their long life cycle and slow growth rate that imply long-term interannual monitoring efforts to detect community changes. Indeed, the few studies on primary succession of coral communities that have been conducted on submerged lava flows have focused on recovery and subsequent changes in community structure^8,33,34^. While volcanic eruptions generally cause important destruction of nearby coral reefs, they also create new substratum for the settlement of reef organisms and increase connectivity, which may induce rapid recovery of reef communities^35^. In Hawaii, Grigg and Maragos^33^ observed that succession appeared to be frequently interrupted, resulting in early successional stages with clearly identified pioneer species. They estimated a recovery period of 20 years for areas exposed to strong hydrodynamic forces, and over 50 years on sheltered ones. Tomascik et al.^34^ investigated early successional dynamics of coral communities following the 1988 volcanic eruption of Gunung Api, in Indonesia. They provided evidence for rapid coral colonization (5 years) and growth on sheltered lava flows, with higher diversity, abundance and cover of coral assemblages than on adjacent carbonate reefs not covered by lava. A rapid recolonization driven by high recruitment of juvenile corals has also been documented four years after the eruption of the Hunga Tonga-Hunga Ha’apai volcanic island^35^. In some cases, the ecological processes driving the recovery dynamics have been examined (e.g., density-dependence^36^), but in the majority of cases, the underlying ecological processes associated to recovery trajectories of coral assemblages, such as recruitment, early mortality and growth, remain elusive, and therefore so do the mechanisms of ecological succession^29,37,38^. In the context of “coral reef crisis” and the pessimistic projections for the future of this ecosystem, examining succession processes is not only important for understanding changes and recovery trajectories of coral communities, but is also critical to better evaluate the potential colonization of climate change refugia, such as non-reef marginal tropical environments or high-latitude temperate regions^39–42^.

Reunion Island (Western Indian Ocean) hosts the Piton de la Fournaise, one of the most active volcanoes characterized by frequent eruptions in recent decades (27 from 1998 to 2007)^43^. Lava flows generated by Piton de la Fournaise often reach the ocean along approximately 20 km of the south-east coastline^44^. These delimited submerged lava flows of different ages offer an ideal field to study the succession of reef communities. Surveys on submerged lava flows of the Piton de la Fournaise have been conducted between 2006 and 2013 by ARVAM (Agence pour la Recherche et la Valorisation Marines), notably during the BIOLAVE expeditions in 2011–2012^45,46^. The structure and diversity of fish^47,48^, echinoderm^49^, marine flora^46^, and ascidian, sponge and octocoral^45^ communities were assessed on lava flows of different ages.

By comparing the diversity, structure (density, cover, colony size) and demography (recruitment, growth, mortality) of coral assemblages among coral reef habitats and lava flow of different ages, we examined the strength and mechanisms of ecological succession for corals, and their agreement to the theoretical assumptions of Connell and Slatyer^1^. Our hypothesis is that the facilitation model should be manifested by species replacement through time and a positive age-diversity relationship, with pioneer species restricted to early stages (Table 1). For the tolerance model, we hypothesize that the sequential change in community composition and diversity would involve a cumulative addition of species, with early succession species also present in older stages. For the inhibition model, early successional stages should be largely monopolized by few pioneer species, which inhibit the recruitment and growth of other species, and with no/few differences in diversity and community composition between early and older successional stages, assuming a similar history of disturbances among sites. Although a long-term monitoring survey including large-scale disturbances would have been ideal to rigorously address theoretical assumptions of ecological succession in coral reefs, such an approach was very difficult to implement in our survey, mainly due to logistical constraints to work at lava flow sites. Consequently, we favoured surveying several descriptors in a restricted period of time, to address important ecological processes such as recruitment and demography, despite the detriment of the temporal variability. By comparing lava flows of different ages, this work still represents a powerful approach to evaluate the predictability of primary succession in highly diverse ecosystems such as coral reefs, while providing valuable insights into the patterns of colonization of new habitats.

**Table 1:** Synthesis of the three major models of ecological succession (as proposed by Connell and Slatyer^1^), their translation for reef coral communities on lava flows of different ages, and confrontation of these models with the results obtained in this study.

Summary of the three models of ecological succession	Expectations of these models for reef coral communities on lava flows of different ages	Main results of our survey in favor (+) or disagreement (−) with the three models
The "facilitation" model: Colonization and growth of the later species is dependent upon the pioneer species "preparing the ground", and being progressively replaced by mid- and late successional species; only after this can later species colonize	Positive age of lava flows-diversity relationship manifested by species replacement through time, with pioneer species restricted to youngest lava flows	− No pioneer species − No relationship between the age of the lava flow and species composition
The "tolerance" model: Later species will be those able to tolerate lower levels of resources than earlier ones; they can invade and grow to maturity in the presence of those that preceded them	Sequential change in community composition and diversity, through a cumulative addition of species, with pioneer species also present in older stages	+ Species richness, abundance and cover of coral assemblages were lower at the youngest lava flow site + Dominance of *Pocillopora* spp. at all lava flow and at most coral reef sites
The "inhibition" model: all species resist invasions of competitors, the pioneer species exclude or inhibit later colonists until the former die or are damaged, thus releasing resources and allowing later colonists to reach maturity	Youngest lava flows largely monopolized by few pioneer species, with no/few differences in diversity and community composition between lava flows of different ages	− No pioneer species − Dissimilarity among lava flow sites

## Material and methods

### Study sites

**Figure 1:**
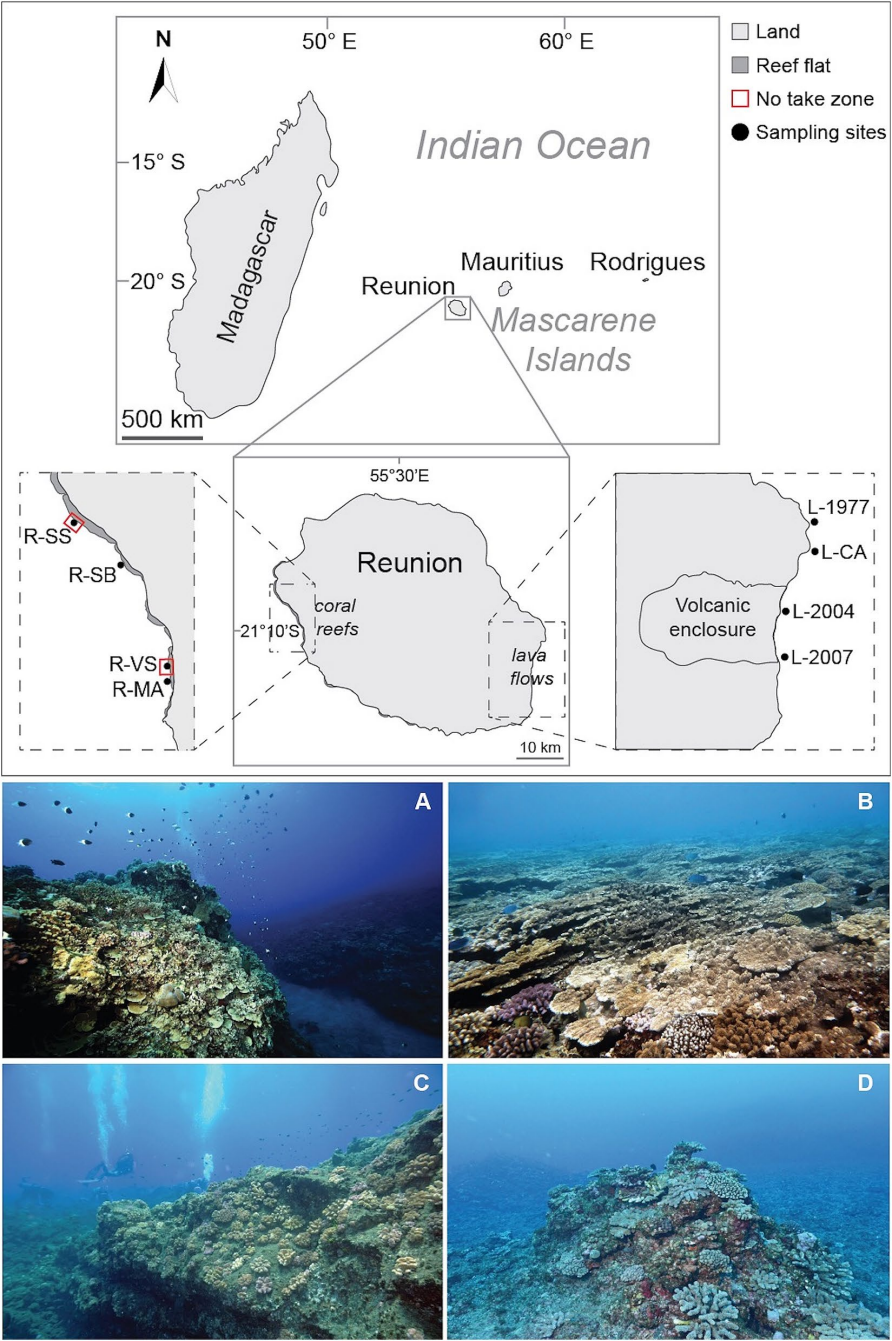
Location of the sampling sites at Reunion Island. Four sites were placed on outer reef slopes of coral reef habitats (coded R-), and four sites on underwater lava flows of different ages (coded L-). R-SS: Sanctuaire Sud; R-SB: Souris Blanche; R-VS: Varangue Sud; R-MA: Marine; L-1977: 1977 lava flow; L-CA: Caesari, a secular lava flow; L-2004: 2004 lava flow; L-2007: 2007 lava flow. The map was created using Adobe Illustrator CS5 (http://www.adobe.com/fr/products/illustrator.html). Photographs showing the contrasted reefscape between coral reef habitat (**A**), with a relatively diversified coral assemblages (**B**), and lava flows (**C**), with a marked dominance of *Pocillopora* spp. (**D**). Photos: Suzac Guenot (**A**), Armand Daydé (**B**), Jean-Pascal Quod (**C**,**D**).

Reunion (21° 07′ S, 55° 32′ E) is a volcanic island (ca. 70 km long and 50 km wide) of the Mascarene Archipelago in the western Indian Ocean, located 700 km east of Madagascar. Fringing reefs line the island in a 12 km^2^ area along 25 km of the west-southwest coast^50^. The west and south coasts of Reunion are subjected to austral swells that occurs throughout the year (with more than 50% from June to September), whereas trade wind swells mainly affect the north, east and south coasts and usually spare the west coast. Cyclonic swells mainly affect the north and east coasts of the island on an ad hoc basis (for example following cyclone Gamède in 2007, Dumile and Bejisa in 2013, Dumazile and Berguitta in 2018). Coral communities are also subject to bleaching events, with the last major ones in 1998, 2002, and 2007^51–53^. Sampling strategy consisted of eight study sites, randomly located at ~ 12 m depth (Fig. 1). Since coral reefs are restricted to the west coast, whereas recent lava flow sites are exclusively found on the southeast coast, we could not place study sites on both habitats within the same area, which is a sampling design (possible at some sites of Hawaiian islands^54^) that would have reduced the potential for environmental factors that can confound comparisons between habitats. Four sites were placed on outer reef slopes of coral reef habitats (coded R-), including two sites (R-SS and R-VS) inside the no-take zone (NTZ) of the marine protected area (Réserve Naturelle Marine de La Réunion; RNMR), and two sites (R-SB and R-MA) outside the NTZ. Additionally, four sites were placed on underwater lava flows of different ages (coded L-): one is a centennial lava flow (L-CA) and the others correspond to more recent eruptive events of 1977 (L-1977), 2004 (L-2004) and 2007 (L-2007). All lava flow sites are made of basaltic substrate resulting from volcanic eruptions, whereas coral reef sites are located on the reef framework, resulting from the prolific growth of calcium carbonate secreting corals. Coral reefs of Reunion Island are about 8000 years old, which is one order of magnitude older than studied lava flows^50^. Fieldwork conducted within the perimeter of the RNMR was realized under authorization N°2014-27 DEAL/SEB/UBIO.

### Sampling strategy

Coral assemblage composition (Scleractinian corals and the calcareous hydrocoral *Millepora*), density, cover, and size-structure were recorded in September 2016 at coral reef sites (R-SS, R-SB, R-VS and R-MA) and in March 2017 at the lava flow sites (L-CA, L-1977, L-2004 and L-2007). Densities of adult and juvenile corals were evaluated on photographs taken along three replicate belt-transects (1 × 10 m) deployed at each site. A mean number of 18 photographs were analysed per belt-transect (we could not take the 20 photos planned per belt-transect at each site, notably at some lava flows where high swell during sampling surveys had forced us to shorten some dives, or when some photos, also at lava flow sites, were not of sufficient quality to interpret). Each coral colony encountered was identified to genus and its maximal diameter measured using a tape present in each photograph using Image J software. At each site, coral cover was evaluated using the Line Intercept Transect method (LIT)^55^ with three replicate linear 20 m transects laid parallel to depth contours and separated by 5 m. As some species may be omitted using belt-transects and LIT^56^, coral species diversity was assessed during a 45 min inspection at each site.

Coral recruitment was characterised through the spatio-temporal variability of the abundance and taxonomic composition of recruits on individual unglazed terracotta tiles attached to a stainless steel support anchored to the substrate^57,58^. For each site, 20 individual tiles (ca. 10 × 10 × 2 cm) were immersed for six months over two summer periods (October 2016–March 2017, October 2017–March 2018). At the end of the immersion periods, tiles were retrieved, bleached and sun dried. The skeletons of coral recruits (coral spats) were then counted and identified. Recruits of Acroporidae, Pocilloporidae and Poritidae families were differentiated according to morphological traits^59^. Other coral recruits were assigned to the category ‘others’, or to the category ‘broken’ when the skeleton was too damaged for identification^58^. Coral recruits found on the different sides of tiles (upper and lower surfaces, and sides) were pooled to estimate mean recruitment density at each site (recruits.m^−2^).

Demographic processes of the three dominant coral genera, *Acropora*, *Pocillopora* and *Porites* (which represent ~ 96% and ~ 70% of the total coral cover on lava flows and reefs, respectively), were evaluated through the survey of juvenile (colonies ≤ 5 cm in diameter) and adult (mature colonies > 5 cm in diameter) stages during one year. At each site, colonies were spatially referenced and measured every six months (two periods: October 2016–March 2017 and April 2017–September 2017) within three replicate belt-transects of 10 m^2^ (1 × 10), laid parallel to depth contours and separated by ~ 1 m. Colony diameter (mm) was used to describe colony size and calculated as the mean between the maximal diameter and its perpendicular. Growth rates (%) and mortality rates (%) were calculated for each colony over the two periods. Mean positive growth rates (${\Delta D}_{+}$) and mean negative growth rates (${\Delta D}_{-}$) were evaluated for each genus-stage combination. To define demographic transitions between two samplings (i.e., 6 months), each coral growth rate $\Delta Di$ was compared to ${\Delta D}_{+}$ and ${\Delta D}_{-}$ associated to the genus-stage combination of the coral $i$. If $\Delta Di>{\Delta D}_{+}$ coral was assigned to positive growth transition, if $\Delta Di<{\Delta D}_{-}$ coral was assigned to negative growth transition, otherwise coral was assigned to retention. Mortality (total loss of a colony living tissues) was also recorded on the field and taken into account as demographic transitions.

### Data analyses

Species composition was compared among sites using a correspondence analysis (*CA* function from *FactoMineR* package for R software^60^), a multivariate analysis based on the chi-square distance. CA is recommended for examining gradients of sites based on species composition, and has the advantage of allowing both species and sites to be displayed on the ordinations axes^61^. Densities, cover (as percentage of space covered), size, recruitment and growth were compared between habitats (coral reefs *vs.* lava flows) and sites using an ANOVA model (see Supplementary Table S1). Spatial factors correspond to a two-level nested design with sites within habitat. For size, recruitment and growth, separated analyses were performed for each selected taxon. These spatial factors correspond to a two-level nested design with sites within habitat. Coral density and size structure were log-transformed and recruitment density was log(x + 1) transformed to meet the assumptions of normality. To identify differences between levels of significant factors, pairwise *t*-tests with Bonferroni correction were used. Coral mortality rates and demographic transitions (positive and negative growth, retention and mortality) were compared among habitats and sites using Fisher’s exact tests. Data analyses were conducted using R software^62^.

## Results

### Coral diversity

**Figure 2:**
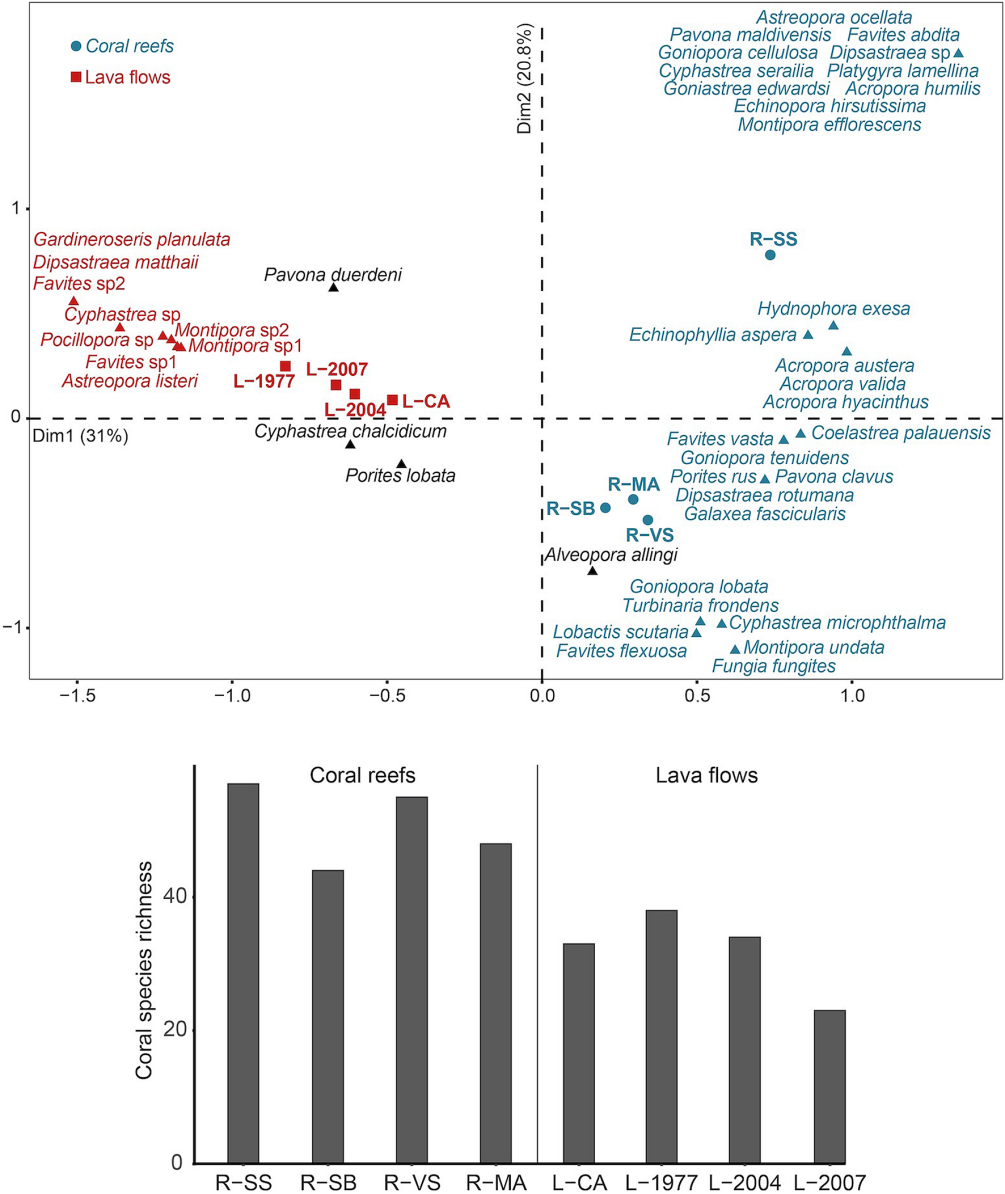
Correspondence analysis showing the spatial variability in the species composition of coral assemblages among sites (**A**), and differences in species richness (**B**) among coral reef and lava flow sites. In blue, species found exclusively on coral reefs; in red, species found exclusively on lava flows; in black, species found in both habitats. One colored triangle may correspond to several species, when this pool of species has the same coordinates on the first two axes. For greater readability, only the 43 species (50% of the species recorded) that contributed the most to the CA are represented. See legend of Fig. 1 for site codes.

A total of 86 species belonging to 33 genera were identified on coral reefs and lava flows (see Supplementary Table S2). Species richness was higher at coral reef sites (44–57 species per site) compared to lava flows (23–38 species per site; Fig. 2). We also found a difference between lava flows of different ages, with 23 species identified on the youngest lava flow (L-2007) and 35 ± 3 species on the older ones. The first axis of the correspondence analysis of coral species composition clearly discriminated lava flow from coral reef sites (Fig. 2). Among the 86 species identified, 11 were recorded exclusively on lava flow sites (*Astreopora listeri*, *Dipsastraea matthaii*, *Gardineroseris planulata*, *Pavona explanulata*, *Cyphastrea* sp., *Favites* sp. 1, and *F.* sp. 2, *Montipora* sp. 1, *M.* sp. 2, *M.* sp. 3 and *Pocillopora* sp.) and 36 species were observed only on coral reef sites. The second axis showed that among coral reef sites, R-SS had a different species composition, with *Goniopora cellulosa*, *Acropora humilis*, *Astreopora ocellata*, *Cyphastrea serailia*, *Echinopora hirsutissima*, *Favites abdita*, *Goniastrea edwardsi*, *Montipora efflorescens*, *Platygyra lamellina*, *Pavona maldivensis* and one species of *Dipsastraea* recorded at this site exclusively. A large number of species were recorded in both habitats, with 12 ubiquitous (i.e., recorded at all eight stations), and 39 species found in at least one reef site and one lava flow site.

### Coral density

**Figure 3:**
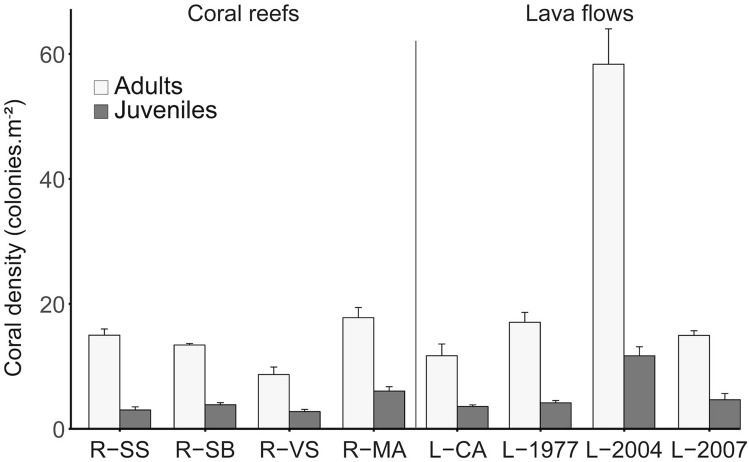
Density of adult (white) and juvenile (grey) coral colonies (all taxa pooled) among coral reef and lava flow sites at Reunion Island (colonies.m^−2^ ± SE). See legend of Fig. 1 for site codes.

A total of 3911 adult and 1069 juvenile colonies were recorded, and adult coral densities ranged from 8.65 (at R-VS) to 58.34 colonies.m^−2^ (at L-2004), while juvenile coral densities ranged from 2.73 (at R-VS) to 11.67 colonies.m^−2^ (at L-2004; Fig. 3). Coral densities for both adults and juveniles were not significantly different between habitats (coral reefs vs. lava flows; ANOVA, p = 0.32 for adults and p = 0.30 for juveniles; see Supplementary Table S1). However, significant differences were detected among sites for both adults and juveniles (ANOVA, all p < 0.0001). Adult density was higher on 2004 lava flow site than at any other site of this habitat (14.5 colonies.m^−2^ in average; pairwise *t*-test with Bonferroni correction, p < 0.001). For coral reef sites, adult density was significantly lower at R-VS compared to the three other sites of this habitat (12.3 colonies.m^−2^ in average; post-hoc tests, p < 0.04). Juvenile density was also significantly higher at L-2004 compared to other lava flow sites (4.1 colonies.m^−2^ in average; post-hoc tests, p < 0.01), and at R-MA (6.0 colonies.m^−2^) compared to other coral reef sites (2.8 colonies.m^−2^ in average; post-hoc tests, p < 0.02). In terms of colony density, coral assemblages at both habitats were generally dominated by *Pocillopora* (43 ± 6%, mean ± SE) and *Porites* (15 ± 4%) and, in a lesser extent, by *Astreopora* (8 ± 3%) and *Acropora* (6 ± 2%) species.

### Coral cover

**Figure 4:**
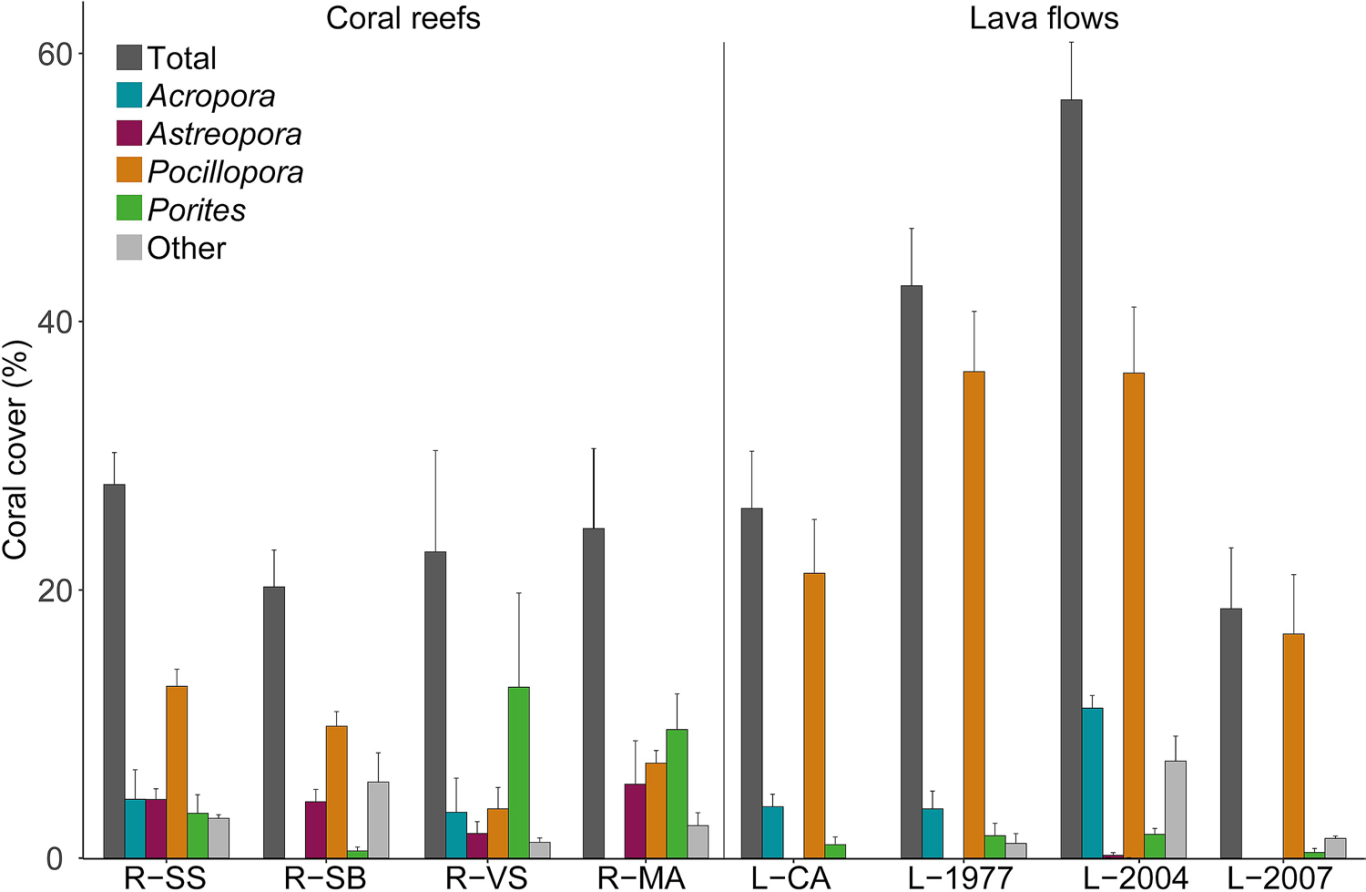
Total coral cover and coral cover of dominant genera (*Acropora*, *Astreopora*, *Pocillopora*, and *Porites*) among coral reef and lava flow sites at Reunion Island (% ± SE). See legend of Fig. 1 for site codes.

Mean coral cover ranged between 20.2 ± 2.7% (mean ± SE) and 27.9 ± 2.4% on coral reef sites, and between 17.8 ± 4.4% and 55.4 ± 4.1% on lava flows, but no significant difference was recorded between habitats (ANOVA, p = 0.23; Fig. 4; see Supplementary Table S1), nor among reef sites (pairwise *t*-test with Bonferroni correction, all p < 0.05). In contrast, a significant difference was noticed among lava flow sites, with higher values at L-2004 and L-1977 compared to L-2007 and L-CA (pairwise *t*-test with Bonferroni correction, p < 0.02). The four genera presenting the highest densities (*Pocillopora, Porites*, *Acropora* and *Astreopora*) were also the most important in terms of coral cover (see Supplementary Table S1 for details of the ANOVA per genera). *Pocillopora* dominated coral assemblages for two of the four coral reef sites (ca. 48.7 ± 12.6% at R-SB and R-SS *vs.* 27.5 ± 15.7% at R-VS and R-MA) and for all lava flow sites (from 64.7 ± 7.5% at L-2004 to 93.9 ± 6.3% at L-2007). While cover of *Porites* could reach high values at coral reef sites (48.4 ± 19.6% at R-VS), this genus was poorly represented at lava flow sites (3.30 ± 0.03%). Similarly, cover of *Astreopora* ranged from 10.9 ± 9.5% to 20.5 ± 4.1% at coral reef sites, but was less than 0.1 ± 0.3% at lava flow sites. For *Acropora*, covers of 14.2 ± 8.0% were recorded at both lava flow (L-2004, L-1977 and L-CA) and coral reef (R-SS and R-VS) sites, whereas coral cover of this genera was null at the R-MA and R-SB coral reef sites and at the youngest lava flow (L-2007).

### Colony size

**Figure 5:**
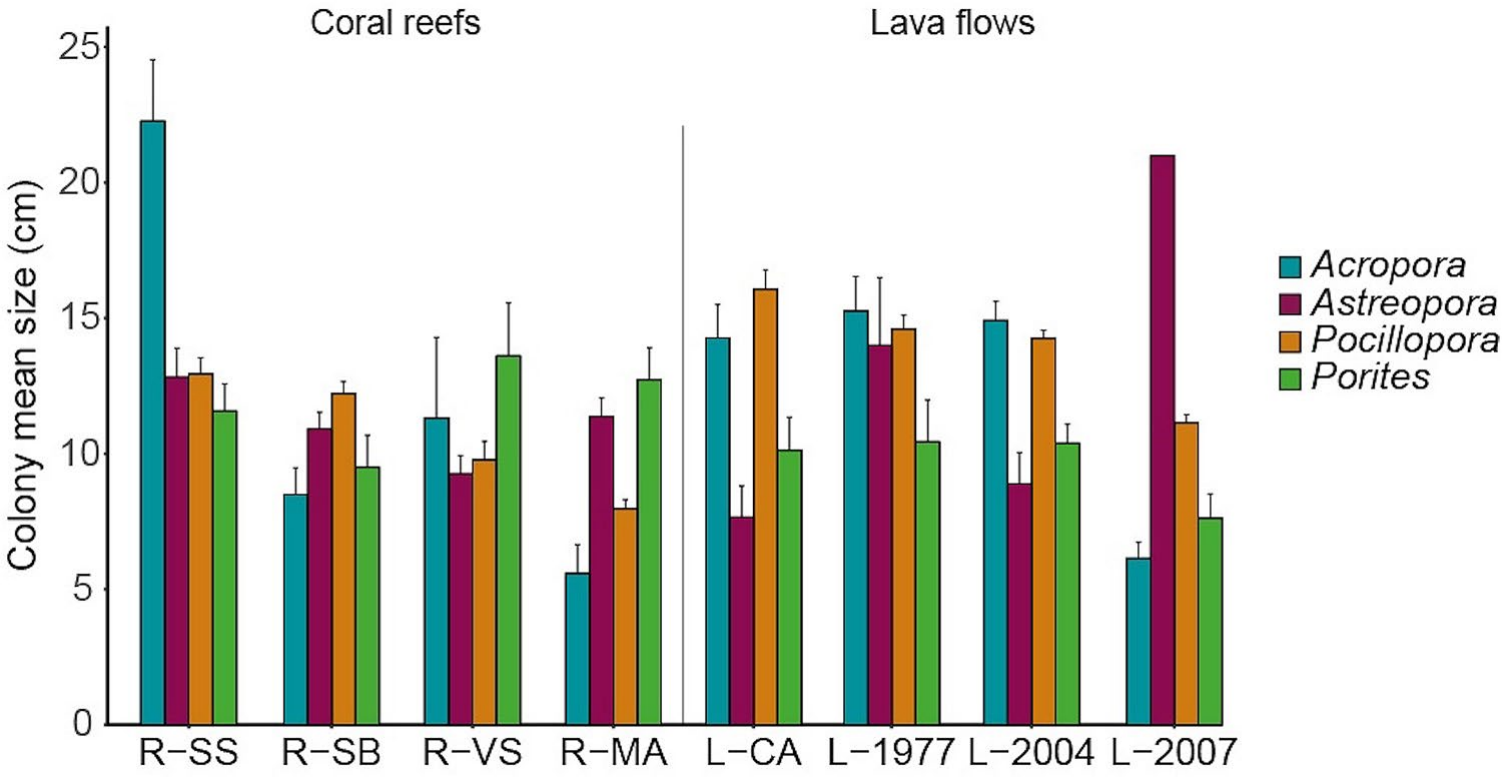
Colony mean size of the dominant coral genera (*Acropora*, *Astreopora*, *Pocillopora*, and *Porites*) among coral reef and lava flow sites at Reunion Island (cm ± SE). Only one *Astreopora* colony was found on the youngest lava flow L-2007. See legend of Fig. 1 for site codes.

Among the 4980 colonies measured, mean colony size of the dominant genera (*Pocillopora, Porites, Acropora* and *Astreopora*) was not significantly different between habitats (ANOVA, all p > 0.1), but significantly varied among sites within habitats for *Acropora*, *Astreopora* and *Pocillopora* colonies (ANOVA, all p < 0.02; Fig. 5; see Supplementary Table S1). At coral reef sites, the mean size of *Acropora* colonies was higher at R-SS (22.3 ± 2.3 cm, mean ± SE) than on the other sites (8.47 ± 2.9 cm; pairwise *t*-test with Bonferroni correction, p < 0.02). The mean size of *Astreopora* colonies was significantly higher at R-SS (12.8 ± 1.1 cm) than at R-VS (9.3 ± 0.7 cm; pairwise *t*-test with Bonferroni correction, p = 0.02), while the mean size of *Pocillopora* colonies was significantly higher at R-SB and R-SS (12.6 ± 0.5 cm) than at R-MA and R-VS (8.9 ± 1.3 cm; pairwise *t*-test with Bonferroni correction, p < 0.01). At lava flow sites, the mean size of *Acropora* and *Pocillopora* colonies were lower on the youngest (2007) lava flow (6.1 ± 0.6 cm and 11.2 ± 0.3 cm, respectively) than the other lava flow sites (14.8 ± 0.5 cm and 14.9 ± 1.0 cm respectively; pairwise *t*-test with Bonferroni correction, p < 0.03). The number of recorded *Astreopora* colonies was too low on the lava flows to allow pairwise comparisons.

### Recruitment patterns

**Figure 6:**
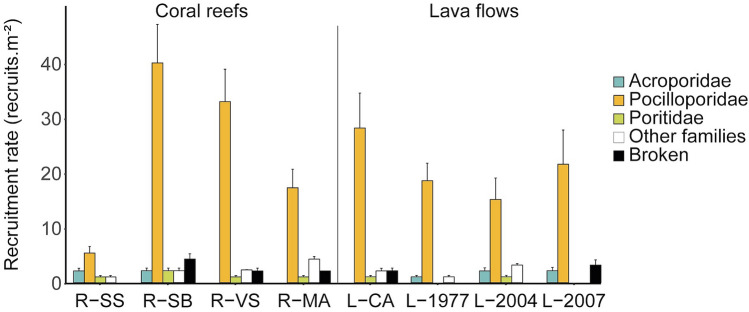
Mean coral recruitment densities of five categories (Acroporidae, Pocilloporidae, Poritidae, Other families, Broken) recorded during two summer periods (October 2016–March 2017 and October 2017–March 2018) on artificial settlement tiles among coral reef and lava flow sites at Reunion Island (recruits.m^−2^ ± SE). See legend of Fig. 1 for site codes.

Over the two studied summer periods (October 2016–March 2017 and October 2017–March 2018), recruit assemblages were largely dominated by Pocilloporidae on both coral reef (77%) and lava flow (83%) habitats (Fig. 6). Acroporidae represented respectively 4% of recruit assemblages in both habitats, Poritidae represented 4% on coral reefs and 2% on lava flows, whereas the other taxa represented 8% and 5%, and the broken recruits 7% and 5%, respectively. Mean densities of coral recruits were not significantly different between reef and lava flow habitats for Acroporidae, Pocilloporidae and Poritidae (ANOVA; all p > 0.05; see Supplementary Table S1). Density of Pocilloporidae recruits was highly variable among coral reef sites (36.5 ± 3.5 recruits.m^−2^, mean ± SE and 5.4 ± 1.2 recruits.m^−2^ at R-SB and R-VS respectively; pairwise *t*-test with Bonferroni correction, p < 0.01), whereas no differences were found among lava flow sites.

### Demographic process

**Figure 7:**
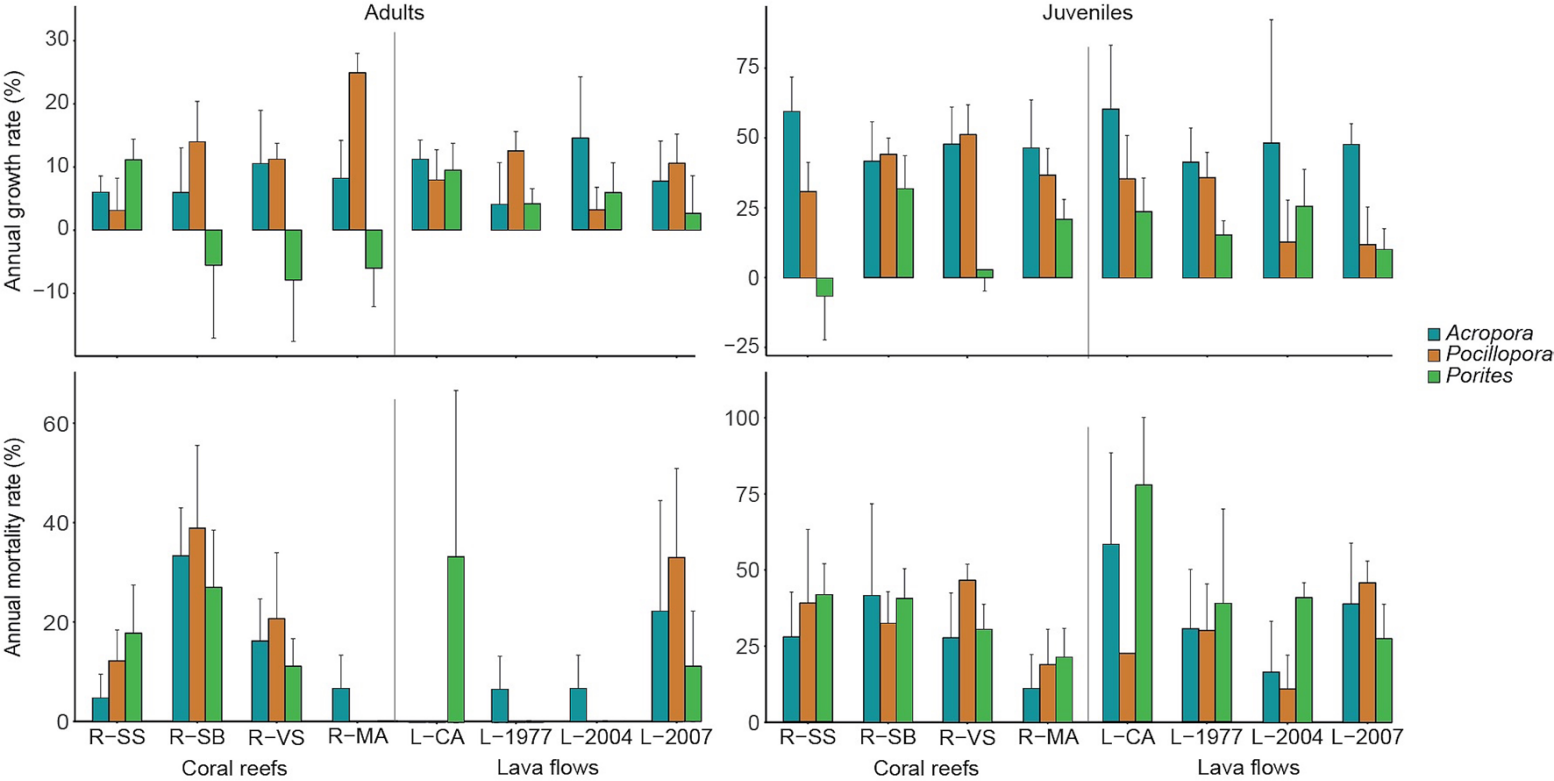
Annual coral growth and mortality rates of adult (left) and juvenile (right) colonies of *Acropora*, *Pocillopora* and *Porites* in coral reef and lava flow sites at Reunion Island (% ± SE). See legend of Fig. 1 for site codes.

**Figure 8:**
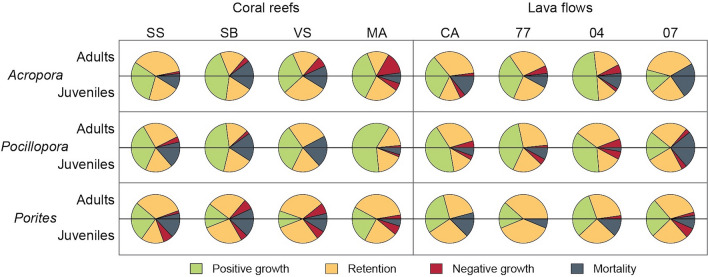
Demographic transitions (Positive growth, Retention, Negative growth, and Mortality) of juvenile and adult colonies of *Acropora*, *Pocillopora* and *Porites* for two 6-months consecutive periods (October 2016–March 2017 and April 2017–September 2017) in coral reef and lava flow sites at Reunion Island. Each circle is divided into two parts (see horizontal black lines), with the upper half circle showing data for adults, and the lower half corresponding to juvenile data. See legend of Fig. 1 for site codes.

Overall, 493 adult and 468 juvenile colonies were sampled. Annual growth rates of the three major coral genera (*Pocillopora*, *Porites* and *Acropora*) did not significantly differ between habitats (coral reef vs. lava flow sites) for adult nor juvenile colonies, except for the juveniles of *Pocillopora* that showed higher growth rates on coral reef sites (ANOVA, p = 0.01; Fig. 7; see Supplementary Table S1). Moreover, growth rates were not significantly different among sites within habitats for any of the three genera (ANOVA, all p > 0.05). Mean growth rate of *Acropora* was higher for juvenile (+ 56%, considering the initial size of the colony, equal to + 17 mm of linear growth) than for adult (+ 8% or + 9 mm of linear growth) colonies. For *Pocillopora*, mean growth rate reached + 10% (+ 11 mm) for adults and + 37% (+ 13 mm) for juveniles. Gain and loss of living tissue were equivalent for adult colonies of *Porites*, resulting in a mean growth rate of + 0.5% (but − 2 mm of mean linear growth), whereas growth rate averaged + 16% for juveniles (+ 4 mm). Except for *Pocillopora* at R-SB, mortality rates were higher for juveniles compared to adults at all sites. No significant differences in mortality rates of *Pocillopora*, *Porites* and *Acropora* colonies were detected between habitats, for adults nor juveniles (Fisher’s exact tests, all p > 0.05; Fig. 7). Mortality of adult *Pocillopora* significantly varied among the four sites of reefs (19 ± 8%, mean ± SE) and those of lava flows (8 ± 8%; Fisher’s exact tests, both p < 0.03). Mortality rates of adult *Acropora* and *Porites* colonies and juveniles of the three genera were not significantly different among sites of both habitats (Fisher’s exact tests, all p > 0.14). Rates of demographic transitions were significantly different between coral reef and lava flow sites for adult *Pocillopora* and *Porites* (Fisher’s exact tests, all p < 0.02; Fig. 8). On coral reef sites, 44% of adult *Pocillopora* colonies showed *positive growth*, and on lava flow sites, 27%. In contrast, *Porites* with *positive growth* represented 31% of their adult population on lava flow sites, and 17% on coral reef sites, where *mortality* was three times higher. Adult colonies of *Pocillopora* also showed significant differences in rates of demographic transitions within habitats, for both lava flow and coral reef sites (Fisher’s exact tests, p = 0.006 and p = 0.003 respectively). For adult *Acropora*, similar within-habitat differences were detected between reef sites (Fisher’s exact tests, p = 0.003).

## Discussion

As in many other coral reefs throughout the world^63–66^, coral assemblages at Reunion Island are characterized by a marked spatial heterogeneity at the local scale (i.e., within reef habitats). Density, cover, colony size, recruitment patterns and mortality of corals show significant differences among sites within coral reefs and lava flows, the two major habitats studied in this survey. Although it was outside the scope of this study, this within-habitat heterogeneity is likely governed by a variety of interacting extrinsic physical and biological drivers, such as the availability of adequate substrate, sediment characteristics, light, water quality, hydrodynamic forces, and biotic interactions^52,67^. Except for demographic transitions of *Pocillopora* and *Porites* adult colonies and for growth rates of *Pocillopora* juvenile colonies, no marked differences between habitats were detected for descriptors of structure and demography of coral assemblages. In fact, the distinction between coral reef and lava flow sites was mostly reflected in the species diversity and composition of coral assemblages, with 11 (out of 86) species exclusively recorded at lava flow sites, and 36 species only observed at coral reef sites. Species richness was always higher at coral reef sites (44–57 species per site) compared to lava flow (23–38 species per site), indicating that maturity of coral assemblages is difficult to reach in this habitat. As proposed for ascidians, sponges and octocorals for which similar patterns were observed^45^, the lower species diversity at lava flow habitats compared to coral reefs may results from different frequency and intensity of perturbations and extremes in environmental conditions. These include higher exposure to cyclonic swells and regional currents on the southeast coasts compared to the west coasts, as well as light availability and sedimentation. In fact, darkness of the black volcanic substrata on lava flows may absorb rather than reflect incident light, thus limiting light availability^45^. Furthermore, the resuspension of volcanic sand and rubble displacement, responsible for smothering and abrading sessile invertebrates, reduces the growth of endosymbiotic organisms such as Scleractinian corals^45^. However, the comparison between habitats is difficult to fully understand in our case, given the distance between the lava flow sites located on the southeast coast and the coral reefs in the northwest, which probably induces confounding factors that may blur such comparison.

Results identified a set of species typical of lava flow habitats, but did not detect a clear relationship between the age of the lava flow and species composition. Even if some “lava flow” species were recorded at contemporary sites (L-2007, L-2004, and L-1977) and not at the older centennial site (L-CA), no strictly pioneer species (e.g., present exclusively on the youngest lava flow) were recorded. These outcomes are in contrast with those obtained at the same location for Echinoderms, for which several pioneer species were exclusively recorded at the youngest lava flow^49^, and for algae, for which species composition was correlated to the age of the lava flows with a high occurrence of pioneer species on recent sites^46^. These differences among reef taxa could be linked to the long life cycle and slow growth dynamics of corals with respect to other organisms, suggesting that successional processes can be very slow for corals.

However, species richness, density and cover of coral assemblages were lower at the youngest lava flow site (L-2007, 10 yo) compared to older ones, where maximal values were recorded at the 1977 and 2004 lava flow sites. Despite the limited number of sites on lava flow habitats, and the limited duration of our survey, these results partly coincide with theoretical assumptions of the facilitation and tolerance models of ecological succession, which predict an increase in species abundance and diversity during early colonization stages, associated with reduced dominance of one or a few species through time^1,7^. After these early successional stages, the relationship between species diversity and ecosystem development becomes less obvious, as large-scale disturbances or local stressors may limit the diversity of mid- and late-successional stages^1,2,6^. Results are also consistent with patterns of coral community succession recorded in Hawaii, where species richness on lava flows of 2–100 yo increased for 45 years and then decreased^33^. This reveals a succession pattern in which colony size and species richness of corals increase over time, the latter due to the settlement of additional species, until species interactions such as competition for space or disturbances lead to their stability or decrease^8,33^. This general pattern has already been observed for Echinoderms^49^ and sessile, soft bodied organisms^45^ on lava flows at Reunion Island, and for deep-water coral communities in Hawaii^54^, but contrasts with those obtained for fish communities for which species richness tended to be higher closer to the most recent lava flow^47,48^.

Results highlight the strong dominance of the genus *Pocillopora*, notably *P. verrucosa* and *P. meandrina*, at all lava flow sites, and at two of the four coral reef sites. This dominance of *Pocillopora* spp. was also recorded during the 2011–2012 BIOLAVE expeditions, notably on the 2007 lava flow (80% of counted colonies). These results indicate that the dominance of *Pocillopora* spp. was established relatively soon (at least 4 years) after the eruption and was maintained for up to 10 years. The opportunistic nature of *Pocillopora* has already been highlighted on natural and artificial reefs^25,38,68,69^ but also over a 1.6 yo basaltic lava flow in Hawaii^33^, and at Hunga Tonga-Hunga Ha’apai island 4 years after a volcanic eruption^35^. However, Tomascik et al.^34^ suggested that the opportunistic nature of species depends on local environmental conditions such as the type of substrate and/or exposure to strong hydrodynamic forces. These authors found that *Acropora* species were the most efficient colonizers on a 5 years old sheltered (in terms of winds and waves) andesitic lava flow, whereas on unstable aggregate of pyroclastic deposits of the same age, *Pocillopora*, *Montipora* and *Porites* were the best colonizers^34^. *Pocillopora* spp. were also found in high abundances on coral reef habitats of the west coast of Reunion Island, being the dominant taxa at some sites. This suggests that *Pocillopora* species group is not only a successful colonist taxa during primary succession, but also an opportunistic taxa due to its life-history traits, such as high growth and recruitment rates, that are advantageous to colonisation and dominance of secondary successional stages^70–73^. Our results clearly match those obtained in Hawaii, where *Pocillopora*, the dominant taxa on lava flows and surrounding habitats, had the highest recruitment rates^33^.

Despite the lack of temporal data, including the effects of large-scale disturbances, our results on diversity, structure and demography of coral assemblages among coral reef habitats and lava flow of different ages represent a unique opportunity to assess, in this region, the validity and strength of the succession models proposed by Connell and Slatyer^1^. The facilitation model is not fully supported by our results, as typical pioneer species were not recorded for corals (Table 1). The fact that species abundance and diversity were lower at the youngest lava flow site compared to older ones, and that early successional species (*Pocillopora* spp. group) were also abundant/dominant on older stages argue in favor of the tolerance model. The dominance of *Pocillopora* in most habitats would also argue in favor of the inhibition model, but as this dominance was not incompatible with a relatively high diversity and abundance of other coral taxa, such as those found in coral reef habitats, this model is not fully supported by our results. In fact, the outcomes of this study suggest that the theoretical sequences of ecological successions are difficult to observe in coral reef ecosystems where recruitment and succession processes are more complex, compared to some terrestrial ecosystems where most of the theoretical assumptions has been developed^3,14,16^. This lack of conformity with the classical theory of succession has been also found for fouling communities in temperate ecosystems^74^, and was attributed to the fact that, compared to terrestrial plants, most marine organisms have a lower ability to modify the substrate, do not have the capacity to store dormant seeds of successional species, and are relatively short-lived. At Reunion Island, as probably in most other coral reefs, this absence of match between observed patterns and theoretical assumptions could be linked to the interferences of high frequency and intensity of large-scale disturbances, such as cyclones and bleaching events, that probably distort or set back the expected successional sequences, and thus prevent coral assemblages to reach maturity states. Unfortunately, even if some surveys on the effects of disturbances such as bleaching events, *Acanthaster* spp. outbreaks, and cyclones have been conducted on some coral reef sites of the western coast^51–53^, no quantitative data on such effects have been collected at any of the lava flow sites, which makes these hypotheses difficult to address. Our results would also need to be complemented by information on coral larval dispersal and connectivity within lava flow sites and across other habitats around Reunion Island, to examine how these processes may drive patterns of succession. Moreover, our study focused on coral assemblages and was conducted several years after the last volcanic eruption, which may have had an impact on the outcomes of this study. It would be interesting to examine coral assemblages and their biotic interactions with other taxa (such as spatial competition, allelopathy and density-dependent mechanisms) during the first months of the recolonization processes, as subtle changes in these early stages may have profound and long-lasting effects on coral dynamics^29,36,75,76^.

## Supplementary information

Supplementary Information.

## Data Availability

Data used in this paper can be requested to the corresponding author.
