# Supplementary material for: Diversity, structure and demography of coral assemblages on underwater lava flows of different ages at Reunion Island and implications for ecological succession hypotheses
Source: Sci Rep. 2020 Nov 30;10:20821. doi: 10.1038/s41598-020-77665-z (PMC7705710; doi:10.1038/s41598-020-77665-z)
Supplement: Supplementary file 1 — Supplementary Information. [file 41598_2020_77665_MOESM1_ESM.pdf]

Diversity, structure and demography of coral assemblages on underwater lava flows of different ages at Reunion Island and implications for ecological succession hypotheses

Florian Jouval,[1,2] Lionel Bigot,[1,2] Sophie Bureau,[1,2] Jean-Pascal Quod,[3] Lucie Penin[1,2], Mehdi Adjeroud[2,4*]

[1]Université de La Réunion, UMR 9220 ENTROPIE, Faculté des Sciences et Technologies, 97744 Saint Denis Cedex 9, La Réunion, France

[2]Laboratoire d'Excellence « CORAIL », Paris, France

[3]Agence pour la Recherche et la Valorisation Marines (ARVAM-Pareto), Technopole de la Réunion, BP 80041, 97491 Sainte Clotilde, La Réunion, France

[4]Institut de Recherche pour le Développement, UMR 9220 ENTROPIE, UPVD, Avenue Paul Alduy 66860 Perpignan, France

*Correspondence and requests for materials should be addressed to M.A. (email: mehdi.adjeroud@ird.fr)

Supplementary Information

Table S1. **Summary of the ANOVA tests to analyse spatial variation of density, percent cover, size-structure, recruitment and annual growth rates of coral assemblages among sites and habitats at Reunion Island.** Df: degrees of freedom.

Variable		Replicates	Factor	Df	Mean Square	F	p value
Density	Adults	Transects	Habitat	1	1.11	1.2	0.32
			Site (in habitat)	6	0.92	30.57	<0.0001
	Juveniles		Habitat	1	0.79	1.27	0.3
			Site (in habitat)	6	0.62	10.98	<0.0001
Percent cover	Total	Transects	Habitat	1	800.42	1.82	0.23
			Site (in habitat)	6	440.11	6.6	<0.01
	Pocillopora		Habitat	1	2221.45	12.61	0.01
			Site (in habitat)	6	176.19	5.43	<0.01
	Porites		Habitat	1	170.93	3.58	0.11
			Site (in habitat)	6	47.96	2.12	0.11
	Acropora		Habitat	1	44.42	1.09	0.34
			Site (in habitat)	6	40.81	7.35	<0.001
	Astreopora		Habitat	1	92.43	25.87	<0.01
			Site (in habitat)	6	3.57	0.74	0.63
Size	*Pocillopora*	Colonies	Habitat	1	35.59	3.53	0.11
			Site (in habitat)	6	10.08	26.59	<0.0001
	Porites		Habitat	1	0.01	0.01	0.95
			Site (in habitat)	6	1.02	1.23	0.29
	Acropora		Habitat	1	2.87	0.62	0.46
			Site (in habitat)	6	4.65	9.48	<0.0001
	Astreopora		Habitat	1	0.01	0.01	0.94
			Site (in habitat)	6	0.91	2.61	0.02
Recruitment rate	Pocilloporidae	Tiles	Habitat	1	2.07	0.16	0.7
			Site (in habitat)	6	12.66	3.37	<0.01
	Poritidae		Habitat	1	0.14	1.97	0.21
			Site (in habitat)	6	0.07	0.22	0.97
	Acroporidae		Habitat	1	1.84E-03	0.01	0.09
			Site (in habitat)	6	0.34	0.92	0.48
Annual growth rate	*Pocillopora* adults	Colonies	Habitat	1	1021.2	1.74	0.24
			Site (in habitat)	6	586.27	2.18	0.05
	Pocillopora juveniles		Habitat	1	2700.6	13.56	0.01
			Site (in habitat)	6	199.1	0.18	0.98
	Porites adults		Habitat	1	1717.4	5.22	0.06
			Site (in habitat)	6	328.79	0.6	0.73
	Porites juveniles		Habitat	1	292.8	0.27	0.62
			Site (in habitat)	6	1095.22	1.23	0.3
	Acropora adults		Habitat	1	7.49	0.17	0.69
			Site (in habitat)	6	43.69	0.13	0.99
	Acropora juveniles		Habitat	1	508.5	0.29	0.61
			Site (in habitat)	6	1774.2	0.54	0.78

Table S2. List of coral species (Scleractinians and the calcareous hydrocoral *Millepora*) recorded at each of the coral reef and lava flow sites at Reunion Island.

Species	Coral reef sites				Lava flow sites			
	R-SS	R-SB	R-VS	R-MA	L-CA	L-1977	L-2004	L-2007
Acanthastrea echinata	√	√	√	√		√	√	√
Acropora abrotanoides	√	√	√	√	√	√	√	
Acropora austera	√		√					
Acropora digitifera	√	√	√	√	√	√	√	√
Acropora gemmifera	√		√	√	√	√	√	
Acropora granulosa	√		√	√	√		√	
Acropora humilis	√							
Acropora hyacinthus	√		√	√				
Acropora valida	√		√					
Alveopora allingi		√	√	√	√			
Astrea annuligera	√	√	√	√	√	√	√	√
Astrea curta				√				
Astreopora listeri					√	√	√	
Astreopora myriophtalma		√	√	√	√	√	√	
Astreopora ocellata	√							
Coelastrea palauensis	√		√	√				
Coscinaraea monile	√	√	√	√	√	√	√	√
Cyphastrea chalcidicum		√	√		√	√	√	√
Cyphastrea microphthalma			√	√				
Cyphastrea serailia	√							
Cyphastrea sp.						√		√
Dipsastraea favus	√	√	√	√	√	√	√	√
Dipsastraea matthaii						√		
Dipsastraea pallida	√	√	√		√			√
Dipsastraea rotumana	√	√	√	√				
Dipsastraea sp.	√							
Dipsastraea speciosa	√	√	√	√	√	√	√	√
Echinophyllia aspera	√	√						
Echinopora gemmacea	√	√	√	√	√	√	√	√
Echinopora hirsutissima	√							
Favites abdita	√							
Favites flexuosa		√	√					
Favites pentagona	√	√	√	√	√	√	√	√
Favites sp. 1					√	√	√	√
Favites sp. 2						√		
Favites vasta	√	√	√					
Fungia fungites			√					
Galaxea fascicularis	√	√	√	√				
Gardineroseris planulata						√		
Goniastrea edwardsi	√							
Goniastrea pectinata	√	√	√	√	√			
Goniastrea stelligera	√		√	√	√			
Goniopora cellulosa	√							
Goniopora lobata		√	√	√				
Goniopora tenuidens	√	√	√	√				

Hydnophora exesa	√			√				
Hydnophora microconos	√	√		√		√		
Leptastrea bottae				√				
Leptastrea pruinosa	√	√	√		√	√		
Leptastrea transversa	√	√	√	√			√	
Leptoria phrygia	√	√	√	√		√		
Leptoseris mycetoseroides	√			√			√	
Lobactis scutaria		√	√					
Millepora exaesa	√	√	√	√	√			
Millepora platyphylla	√	√	√	√	√	√	√	√
Montipora efflorescens	√							
Montipora sp. 1					√	√	√	
Montipora sp. 2					√	√		
Montipora sp. 3							√	
Montipora tuberculosa				√				
Montipora undata			√					
Montipora venosa	√	√	√	√			√	√
Paramontastraea peresi		√	√	√		√	√	
Pavona clavus	√	√	√	√				
Pavona duerdeni	√				√	√	√	√
Pavona explanulata							√	
Pavona maldivensis	√							
Pavona varians	√	√	√	√		√	√	
Pavona venosa	√	√	√	√	√	√		√
Platygyra daedalea	√	√	√	√	√	√	√	
Platygyra lamellina	√							
Platygyra pini	√	√	√	√		√		√
Plesiastrea versipora		√						
Pocillopora damicornis			√					
Pocillopora grandis	√	√	√	√	√	√	√	√
Pocillopora meandrina	√	√	√	√			√	
Pocillopora sp.						√	√	√
Pocillopora verrucosa	√	√	√	√	√	√	√	√
Pocillopora woodjonesi			√					
Porites lobata		√	√	√	√	√	√	√
Porites lutea	√	√	√	√	√	√	√	√
Porites rus	√	√	√	√				
Porites solida	√		√	√	√	√	√	
Psammocora profundacella	√	√	√	√	√	√	√	√
Turbinaria frondens		√	√	√				
Turbinaria mesenterina	√		√		√	√		
Total	57	44	55	48	33	38	34	23
